# Conformational specificity of the C4F6 SOD1 antibody; low frequency of reactivity in sporadic ALS cases

**DOI:** 10.1186/2051-5960-2-55

**Published:** 2014-05-14

**Authors:** Jacob I Ayers, Guilian Xu, Olga Pletnikova, Juan C Troncoso, P John Hart, David R Borchelt

**Affiliations:** Department of Neuroscience, Center for Translational Research in Neurodegenerative Disease, McKnight Brain Institute, University of Florida, Box 100159, Gainesville, FL 32610 USA; Department of Pathology, The Johns Hopkins University School of Medicine, Baltimore, MD USA; Department of Biochemistry and X-ray Crystallography Core Laboratory, University of Texas Health Science Center, San Antonio, USA; Department of Veterans Affairs, Geriatric Research, Education, and Clinical Center, South Texas Health Care System, San Antonio, TX USA

**Keywords:** Superoxide dismutase 1, Amyotrophic lateral sclerosis, C4F6 epitope, Conformational antibodies

## Abstract

Greater than 160 missense mutations in copper-zinc superoxide dismutase-1 (SOD1) can cause amyotrophic lateral sclerosis (ALS)*.* These mutations produce conformational changes that reveal novel antibody binding epitopes. A monoclonal antibody, clone C4F6 - raised against the ALS variant G93A of SOD1, has been identified as specifically recognizing a conformation shared by many ALS mutants of SOD1. Attempts to determine whether non-mutant SOD1 adopts a C4F6-reactive conformation in spinal tissues of sporadic ALS (sALS) patients has produced inconsistent results. To define the epitope recognized by C4F6, we tested its binding to a panel of recombinant ALS-SOD1 proteins expressed in cultured cells, producing data to suggest that the C4F6 epitope minimally contains amino acids 90–93, which are normally folded into a tight hairpin loop. Multiple van der Waals interactions between the 90–93 loop and a loop formed by amino acids 37–42, particularly a leucine at position 38, form a stable structure termed the β-plug. Based on published modeling predictions, we suggest that the binding of C4F6 to multiple ALS mutants of SOD1 occurs when the local structure within the β-plug, including the loop at 90–93, is destabilized. In using the antibody to stain tissues from transgenic mice or humans, the specificity of the antibody for ALS mutant SOD1 was influenced by antigen retrieval protocols. Using conditions that showed the best discrimination between normal and misfolded mutant SOD1 in cell and mouse models, we could find no obvious difference in C4F6 reactivity to spinal motor neurons between sALS and controls tissues.

**Electronic supplementary material:**

The online version of this article (doi: 10.1186/2051-5960-2-55) contains supplementary material, which is available to authorized users.

## Introduction

Amyotrophic lateral sclerosis (ALS) is a fatal neurodegenerative disease characterized by the loss of upper and lower motor neurons. Approximately 90% of the cases are sporadic (sALS) in origin whereas 10% are familial (fALS) and caused by mutations identified in more than 10 different genes {http://alsod.iop.kcl.ac.uk/}. One of the first dominantly-inherited fALS-associated genes to be identified was the *SOD1* gene; mutations in *SOD1* account for 10-20% of all fALS cases. There are now more than 160 missense mutations within this gene that have been described in ALS patients {http://alsod.iop.kcl.ac.uk/}. On the basis of studies in various animal models, it is thought that the mutations in *SOD1* cause a gain of toxic properties to produce the progressive paralytic symptoms observed in fALS patients. Importantly, the symptoms and CNS pathology observed in patients harboring SOD1 mutations are very similar to those observed in non-inherited forms of disease, suggesting that there could be related mechanisms of pathogenesis.

The toxic properties of mutated SOD1 are thought to arise from mutation-induced conformational changes leading to SOD1 misfolding and aggregation. Wild-type SOD1 (WT) can acquire some of the same properties as mutant SOD1 when oxidized and stripped of metal cofactors; these preparations have also been shown to be toxic when administered to cells [1–5]. Indeed, transgenic mice that are homozygous for WT SOD1 transgenes and expressing very high levels of protein form aggregate pathology similar to what is seen in mutant SOD1 mice with paralytic symptoms [6–8]. Additionally, co-expression of WT SOD1 with mutant SOD1 almost invariably accelerates the onset of paralysis with evidence that WT SOD1 has been induced to aggregate with mutant SOD1 [9–13]. These studies point to WT SOD1 as a potential pathogenic link between fALS and sALS and more importantly, implicate SOD1 as a target for therapeutic intervention in the majority of ALS cases. Through the course of these studies, conformation-specific antibodies to SOD1 have emerged as critical reagents to distinguish misfolded, presumably toxic SOD1, from protein that achieves a more native conformation. Examples of these antibodies include a series of monoclonal antibodies generated by immunizing mice with a recombinant apo form of G93A hSOD1, yielding antibodies designated C4F6, A5C3, and D3H5 [14]. To date, however, the epitope recognized by these antibodies has not been completely characterized.

The anti-hSOD1 antibody, C4F6, which has been widely used in studies to identify misfolded SOD1, was reported to show strong immunoreactivity to denatured G93A, significant reactivity (but much lower) to other hSOD1 mutants, and very low reactivity to denatured WT hSOD1 [14]. WT hSOD1 can be induced to bind C4F6 by oxidation in vitro, and such reactivity was linked to sporadic ALS by demonstrating C4F6 immunoreactivity to spinal motor neurons in sALS cases [5]. However, when Brotherton et al. used C4F6 to stain spinal cord tissue from sALS cases and an A4V fALS case, they observed that C4F6 reacted with inclusions in the A4V case but not in the sALS cases [15]. More recently Saxena et al. [16] linked the accumulation of A5C3 reactive mutant SOD1 to motor neuron toxicity in the G93A mouse model of ALS. With the emergence of these antibodies as important research tools, and the possible development of these antibodies as therapies [17], it is increasingly important to better understand the nature of the conformational epitope recognized by these antibodies.

The only information currently available on the nature of the epitope is that it is located in exon 4 of the hSOD1 protein [18], which comprises amino acids 80–119 of SOD1. We use a combination of immunohistochemical and biochemical techniques to demonstrate that amino acids 90–93 of the hSOD1 protein, which comprise a loop domain between β-strands 5 and 6, are critical components of the C4F6 epitope. The key residues identified in the epitope include an aspartic residue at position 90 that begins a sequence of Asp-Lys-Asp and position 93 (Ala favored but Gly tolerated). A comparison of X-ray crystal structures of WT and G93A SOD1 did not reveal an obvious conformational change in the 90–93 loop element that could produce its apparent conformational specificity. Instead, our data fit best with a model in which any mutation or modification that increases flexibility in the 90–93 loop enables C4F6 reactivity. Furthermore, we systematically assess the impact of standard antigen retrieval procedures on the immunoreactivity of C4F6, and related antibodies, towards mutant and WT SOD1 in fixed tissue specimens from transgenic mice, finding that standard antigen retrieval techniques greatly influence reactivity. Using conditions optimized in the mouse tissues, we examined the frequency of reactivity with this antibody in post-mortem spinal cord tissue from 25 sALS cases. In our hands, none of the sALS cases we examined showed reactivity that was distinct from what could be observed in controls.

## Materials and methods

### Transgenic mice

All the strains of transgenic mice used in this study have been previously described: WT hSOD1 over-expressing mice used here were the B6SJL-Tg(SOD1)2Gur/J hybrid line [19], G93A hSOD1 in B6SJL-TgN(SOD1-G93A)1Gur mice [19], G37R hSOD1 in Gn.G37R Line29 mice [20], and L126Z hSOD1 in the L126Z Line45 mice [21]. These mice were all maintained in a hybrid background of C57BL/6 J and C3H/HeJ. All studies involving mice were approved by the Institutional Animal Care and Use Committee at the University of Florida. For identification of genotype, DNA was extracted from mouse tail biopsies and analyzed by PCR as previously described [12].

### Subjects

Tissues from sALS cases were collected with patient consent and handled under protocols approved by the Johns Hopkins Institutional Review Board. All samples were coded and de-identified. Spinal cord tissues from a total of 25 sALS cases and 5 non-disease controls were made available for analysis (Additional file 1: Table S1 provides details on the patients and tissues analyzed). Spinal cord tissues were preserved by immersion fixation in 10% formalin for at least 14 days and then processed for paraffin embedding and sectioning at 10 μm. Tissues from ALS cases were analyzed for the presence of relevant pathologic features including motor neuron loss, Bunina body inclusions, Cystatin C positive neuronal inclusions, skein-like inclusions, and spherical inclusions. For these pathologies a semi-quantitative assessment of abundance was used. We also determined which cases showed corpora amylacea inclusions (presence + or absence -).

### Transgenic mouse tissue collection

Spinal cords were collected from mice for immunohistochemistry (IHC) analysis. Animals were anesthetized with isoflurane and perfused transcardially with 20 ml of phosphate-buffered saline followed by 20 ml of 4% paraformaldehyde. Spinal cords were immediately removed and placed in 4% paraformaldehyde for 24–48 hours at 4°C prior to paraffin processing.

### Immunohistochemistry and periodic acid Schiff staining

To characterize the pathologic features of human sALS tissues, 10 μm sections were immunostained with ubiquitin (Rabbit anti-ubiquitin (1:500) from DAKO, CA), which revealed skein-like inclusions, cystatin-C (Rabbit anti-cystatin C (1:100) from Millipore/Upstate Biotech, Catalog #ABC20., Billerica, MA), which revealed Bunina-like inclusions, or by H&E staining, which revealed Lewy- body-like inclusions and Bunina body inclusions. All staining of human tissues used a steam/citrate buffer antigen retrieval protocol. Immunohistochemistry on mouse tissues was performed on 5–7 μm sections. Sections were deparaffinized and either left in water, incubated in 95% formic acid for 20 minutes followed by overnight incubation in 6 M guanidine hydrochloride at room temperature, or steamed in 10 mM citrate buffer with 0.05% Tween 20, pH 6.0 for 30 minutes. Sections were blocked of endogenous peroxidases by immersion in 0.3% H_2_O_2_ in PBS following multiple PBS washes for the sections that underwent antigen retrieval. Following blocking of non-specific staining with 10% normal goat serum in PBS containing 0.5% Tween-20 (PBST), sections were incubated overnight at 4°C with either the C4F6 antibody (Medimabs, Montreal, Quebec, Canada) at a 1:500 dilution or the SEDI antibody (kind gift from Janice Robertson) at a 1:250 dilution in PBST with 3% normal goat serum. The sections were then incubated with a biotinylated secondary anti-mouse antibody (Vector Laboratories, Burlingame, CA) diluted 1:500 in PBST with 3% normal goat serum followed by incubation with the ABC-horseradish peroxidase staining kit (Vector Laboratories, Burlingame, CA). Sections were developed using 0.05% w/v 3,3’-diaminobenzidine (Sigma-Aldrich, St. Louis, MO) in PBS containing 0.0015% H_2_O_2_ and counterstained with hematoxylin. Images were taken using an Olympus BX60 microscope.

Tissue sections were stained with periodic acid Schiff by first oxidizing them in 0.5% periodic acid (Fisher Scientific, Pittsburgh, PA) solution in water for 5 minutes. Following a rinse in distilled water sections were placed in Schiff’s reagent (Sigma-Aldrich, St. Louis, MO) for 15 minutes. Sections were washed in warm tap water for 5 minutes and then counterstained in hematoxylin for 1 minute and washed again in water for 5 minutes. Slides were then dehydrated and coverslipped.

### SOD1 cDNA expression plasmids, cell lines, and transfections

WT and mutant hSOD1 untagged proteins were expressed from plasmids based on the mammalian pEF-BOS expression vector, and have been previously described [11, 22–26]. YFP tagged SOD1 cDNA variants were created from a worm expression vector (pPD30.38) that contains WT hSOD1 fused to eYFP (yellow fluorescent protein) kindly provided by Dr. Rick Morimoto (Northwestern University). Mutant fluorescently tagged SOD1 variants were constructed following similar procedures and have been previously described [12, 27]. All cDNA genes and pEF-BOS vectors encoding these cDNAs were verified by sequencing prior to their use in experimentation. CHO cells (ATCC, Manassas, VA) were used for all cell culture studies in which immunocytochemistry (ICC) was to be performed, and HEK293FT cells (Invitrogen, Carlsbad, CA) were used if biochemical analysis was to be performed. All cell lines were maintained following ATCC recommendations.

Transfection of cells for ICC was performed on glass coverslips that were previously coated with 0.5 mg/ml poly-L-lysine in 1× phosphate buffered saline solution (PBS). A total of 0.8 μg of vector DNA was transfected per well, using Lipofectamine 2000 (Invitrogen, Carlsbad, CA). For biochemical analyses, a total of 4 μg of vector DNA was used to transfect cells in 60 mm poly-L-lysine coated dishes (BD Biosciences, Bedford, MA). Each transfection experiment was repeated a minimum of 3 times.

### Immunocytochemistry

After rinsing the cells 3 times in 1× PBS, transfected cells were fixed with 4% paraformaldehyde in 1× PBS for 15 minutes. Cells were then permeabilized using ice-cold 100% methanol for 5 minutes followed by incubation in 20% normal goat serum in 1× PBS. Immunostaining was then performed with hSOD1 (1:1000) and C4F6 (1:1000) antibodies in 1× PBS with 10% normal goat serum and incubation overnight at 4°C. Cells were then incubated for 1 hour with secondary antibodies (Invitrogen, Carlsbad, CA) Alexafluor goat anti-rabbit 568 for hSOD and Alexafluor goat anti-mouse 568 for C4F6 diluted 1:2000 in 1× PBS with 10% normal goat serum. Cells were treated with 4’,6-diamidino-2-phenylindole, dihydrochloride, stock 14.3 mM (DAPI) (Invitrogen, Carlsbad, CA) diluted 1:2000 in 1× PBS for 10 minutes to visualize nuclei. Fluorescence was visualized on an epifluorescence Olympus BS60 microscope.

### Recombinant SOD1 expression and purification

Recombinant hSOD1 proteins were expressed and purified as previously described [28].

### Dot-blot with recombinant SOD1 protein

Recombinant hSOD1 proteins (1.5 μg), as indicated in figure legends, were spotted onto a nitrocellulose membranes. The proteins were allowed to dry on the membrane for 30 minutes at 25°C and then the membrane was submerged in PBS for 5 minutes. The membranes were then incubated in 4% paraformaldehyde for 30 minutes followed by 5 washes in PBS for 5 minutes each. The membranes were then treated with varying conditions: 1) submerged in 6 M guanidine hydrochloride for 30 minutes or 2) submerged in 10 mM citrate buffer with 0.05% Tween 20, pH 6.0 and steamed for 30 minutes. After each treatment membranes were again washed in PBS 5 times for 5 minutes each. Odyssey blocking buffer (LI-COR, Lincoln, NE) was then used to block the membranes for 1 hour. Immunostaining was performed by incubating the membranes in a solution of C4F6 (1:1000) and hSOD (1:2500) antibodies diluted together in Odyssey blocking buffer with 0.1% Tween 20 at 4°C overnight. Membranes were rinsed in PBST 5 times for 5 minutes each and then incubated in a solution containing both the IRDye 680RD goat anti-mouse and IRDye 800CW goat anti-rabbit antibodies (LI-COR, Lincoln, NE) diluted in Odyssey blocking buffer containing 0.1% Tween 20 and 0.01% sodium dodecyl sulfate for 45 minutes at room temperature. Membranes were then rinsed 4 times in PBS-T for 5 minutes each, 1 time in PBS for 5 minutes, and then imaged using the Odyssey Infrared Imaging Systems (LI-COR, Lincoln, NE). Densitometric analysis was performed using Odyssey software version 3.0.

### Immunoblotting

Following transient transfection for 48 hours with SOD1 constructs as described above, cells were harvested in PBS with 1:100 v/v protease inhibitor cocktail (Sigma, St. Louis, MO). The cells were then lysed by sonicating the samples two times for 15 seconds each before low speed centrifugation (~800 × g) for 10 minutes. Protein concentrations of the supernatant were then determined by bicinchoninic acid assay as described by the manufacturer (Pierce Biotechnology, Rockford, IL). Various protein concentrations, as indicated in figure legends, were boiled for 5 minutes in Laemmli sample buffer with β-mercaptoethanol and electrophoresed in 18% Tris-Glycine gels (Invitrogen, Carlsbad, CA). Following transfer, membranes were blocked in Odyssey blocking buffer (Odyssey) and subsequent processing and imaging using the Odyssey Infrared Imaging Systems (LI-COR) was performed as described for the recombinant dot-blots.

### Molecular modelling

The conformations (rotamers) adopted by the side chains of residues E40, D90, K91, D92, and G93 for WT SOD1 were visualized by superimposing 7 structures of 32 subunits that are available in the protein data bank (PDB) (Additional file 2: Table S2) using PyMOL (The PyMOL Molecular Graphics System, Version 1.7.0, Schrödinger, LLC). The structural consequences of mutation of G93 to A was examined in silico by superimposing 3 structures of 16 subunits for G93A SOD1 that are available in the PDB bank (Additional file 2: Table S2) using PyMOL.

### Statistical analysis

All statistical analyses were analyzed in GraphPad PRISM 5.01 Software (la Jolla, CA) as explained in figure legends.

## Results

### Characterization of the C4F6 epitope

**Figure 1:**
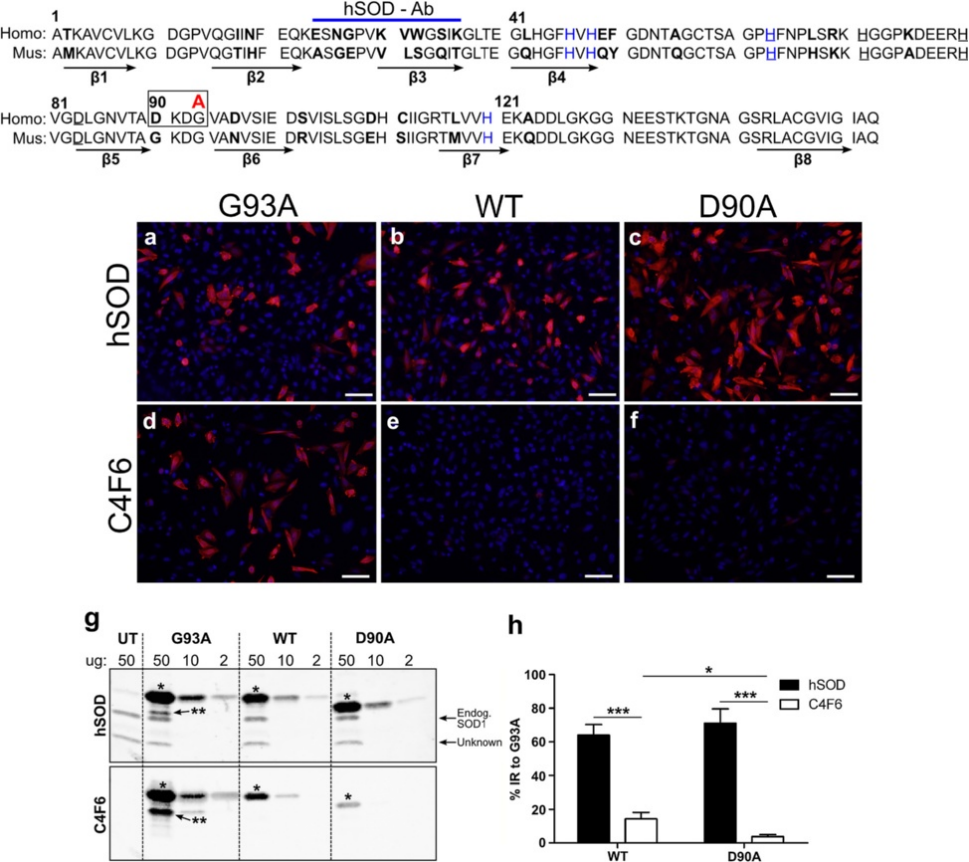
**The D90A ALS mutant binds C4F6 weakly.** Transiently-transfected cells expressing hSOD1 proteins G93A **(a, d)**, WT **(b, e)**, or D90A **(c, f)** were immunostained with either hSOD antibody or C4F6 antibody. Nuclei were stained with DAPI (blue). **(g)** For immunoblot analysis, HEK293FT cells were transiently transfected with G93A, WT, D90A, or left untransfected (UT) for 48 hours and 50, 10, and 2 μg of total protein from homogenates were analyzed by SDS-PAGE. **(h)** Immunoblots similar to those shown in **(g)** were quantified. At each protein concentration, the most intense band (asterisk) was quantified and the intensity was normalized to the intensity for the band produced in cells expressing the G93A variant. The positions of endogenous SOD1 from CHO cells is marked as is the position of an unknown cross-reactive band. In cells transfected with constructs for G93A SOD1, a second faster migrating band was detected by both hSOD1 and C4F6 antibody (double asterisk). This band may be a cleavage product of G93A SOD1 or represent protein modified in some manner. Data from 3 replicate experiments were quantified and graphed (mean ratio ± S.E. (error bars)). *Scale bar* 50 μm. * P ≤ 0.05, *** P ≤ 0.001 (unpaired *t*-test).

From previous characterization of the C4F6 antibody, it had been established that it does not recognize mouse SOD1 [5]. In the sequences adjacent to the G93A residue, there is a sequence difference at position 90, which in human is D and in mouse is G. A mutation of the D at 90 to alanine causes ALS and has been previously shown to cause the protein to aggregate [22]. We therefore, tested the immunoreactivity of C4F6 to the D90A hSOD1 mutation following transient transfection and immunocytochemistry. Robust expression of D90A, along with G93A and WT SOD1 was indicated by immunostaining with the control hSOD antibody that can detect all variants of hSOD1 (Figure 1a-c). However, C4F6 immunoreactivity was absent in cells transfected with both D90A and the control WT hSOD1 (Figure 1e,f). As expected G93A showed strong staining with C4F6 (Figure 1d). This finding indicates that the aspartic acid at amino acid 90 is a critical component of the C4F6 epitope.

To further demonstrate the importance of this amino acid, we analyzed immunoblots of cell lysates containing the WT, G93A, and D90A proteins separated by denaturing SDS-PAGE using the hSOD antibody for assessing protein loading and the C4F6 antibody to determine relative affinity (Figure 1). Three different amounts of cell lysate were loaded on the gel. All three hSOD1 proteins revealed a strong signal when probed with the hSOD antibody, with decreasing intensity of reactivity when less protein was loaded (Figure 1g, asterisk marks position of primary gene product). The D90A variant exhibits slightly faster electrophoretic mobility in SDS-PAGE because the loss of the charged residue increases SDS-binding to the protein [29]. Using fluorescently-labeled secondary antibodies and the Odyssey Imaging System (see Materials and methods), the same blot was then probed with the C4F6 antibody. Although the G93A protein displayed high immunoreactivity for C4F6, the WT and D90A proteins were less reactive (Figure 1g). When normalized to the signal intensity of G93A, the affinity of C4F6 for both the WT and D90A hSOD1 proteins was 5 or 25-fold lower, respectively (Figure 1g,h). Together with the immunocytochemical studies, these outcomes provide strong evidence that the Asp at position 90 is an essential residue in the epitope for C4F6. Considering also that reactivity for denatured G93A SOD1 is much higher than that for denatured WT SOD1, we also conclude that the epitope extends to position 93. Thus, the minimal epitope for C4F6 is predicted to be D-K-D-G/A. Importantly, C4F6 possesses reactivity to the WT sequence of DKDG and thus could react with SOD1 encoding mutations other than G93A if the only consequence of the mutation was to expose the DKDG sequence to allow antibody binding.

### Molecular modeling of the C4F6 epitope

**Figure 2:**
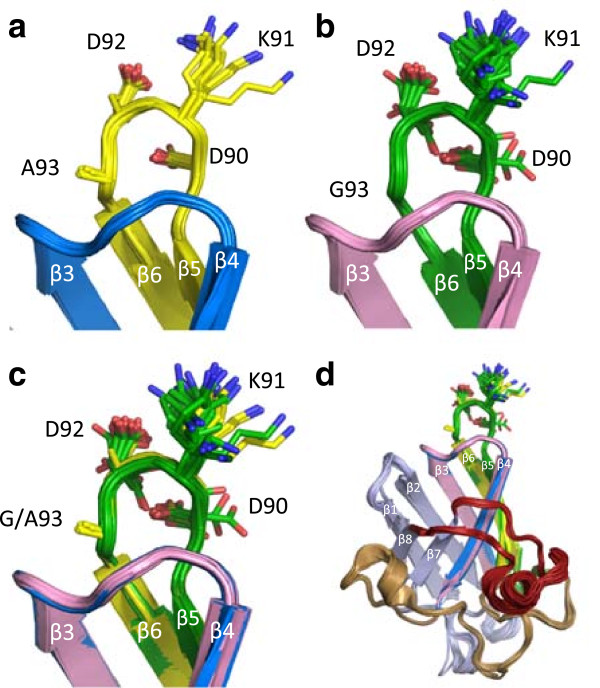
**Structure of WT and G93A hSOD1 in the critical C4F6 binding site. (a)** The critical amino acids thought to be involved in C4F6 binding are located in a loop structure formed by amino acids 90–93. **(b)** Alignment of structures of G93A hSOD1from 3 different crystals with 16 subunits in the PDB database. **(c)** Merged alignment of WT and G93A hSOD1. **(d)** View of aligned complete structures for WT and G93A hSOD1.

To better understand the structure of hSOD1 around this stretch of amino acids and to determine the structural consequences of mutations that affect the C4F6 epitope, we aligned the crystal structures of 32 subunits from 7 distinct crystal structures that are in the PDB database (Additional file 2: Table S2) and merged them into one molecule to reveal the structural heterogeneity of our region of interest (Figure 2a). A striking observation was the high degree of alignment that could be obtained in these structures despite the fact that different crystallization conditions were used to obtain these data. Amino acids 90–93 are located in the beta-turn structure of the loop between the 5^th^ and 6^th^ beta sheets of hSOD1 and are in contact with the loop between the 3^rd^ and 4^th^ beta-sheets (Figure 2a). In the WT structure, the side chain of the lysine at position 91 is highly solvent exposed and can be found in multiple backbone independent rotamers (81 possible; Figure 2a). To determine how the G93A mutation may alter structure in the 90–93 loop, we performed the same type of structure alignments for the available structures of the G93A mutant (16 subunits from 3 distinct crystal structures; Figure 2b). The overall architecture of the G93A variant is very similar to that of WT SOD1, particularly in the loop region of 90–93 (Figure 2b). Superimposition of the structures of WT and G93A shows that the overall conformation of the 90–93 loop, and the adjacent loop between β-strands 3 and 4, in the G93A variant was not obviously different from that of WT (Figure 2c and d).

### Condition-specific immunoreactivity of C4F6 to hSOD1 variants

**Figure 3:**
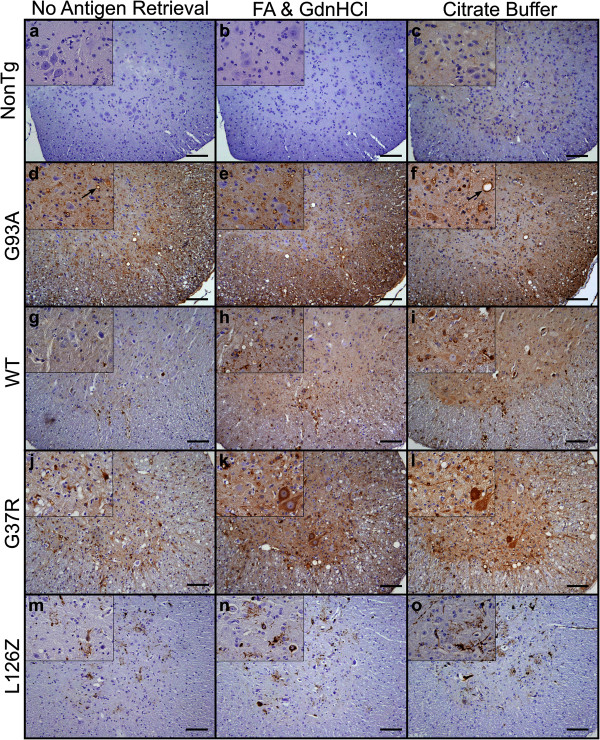
**(a-o) As noted in the margins of the figure, tissue sections from mice overexpressing WT, G93A, G37R, and L126Z hSOD1, or nontransgenic mice for controls, were stained with C4F6 following either no antigen retrieval, formic acid and 6 M guanidine hydorchloride (FA & GdnHCL), or steaming in citrate buffer.** All tissues from mice expressing mutant hSOD1 (G93A, G37R, and L126Z) were harvested from paralyzed mice. The WT hSOD1 tissue was from mice ~18 months of age. Arrows in panels **d** and **f** highlight staining around the vacuolar structures commonly present in G93A mice. *Scale bar* 100 μm.

To determine the utility of this antibody in tissue preparations, we tested the immunoreactivity of the antibody on spinal cord tissue from several lines of transgenic mice expressing hSOD1 variants following different antigen retrieval techniques. We compared the C4F6 immunoreactivity in 4 different lines of hSOD1 expressing mice: the Gurney WT line which display no clinical symptoms but exhibit some of the vacuolar changes seen in G93A mutant mice [30], the G93A line of mice that succumb to disease in 5–7 months of age (in our colony) [19], the G37R Line 29 mice which develop disease in 7–9 months of age, and the L126Z Line 45 mice that express a truncated hSOD1 and succumb to disease at 8–10 months of age. Our forgoing immunoblotting data suggest that denaturation of SOD1 protein could reveal the epitope required for C4F6 immunoreactivity. To test this hypothesis, we employed a treatment with formic acid to break the protein crosslinks formed from fixing the tissue with paraformaldehyde followed by an overnight incubation in 6 M GdnHCl to denature the protein. Immunoreactivity to the SEDI antibody that recognizes an epitope buried in the SOD1 dimer interface was used on the same tissue as a positive control [31]. This antigen retrieval technique was observed to greatly increase the reactivity of the SEDI antibody, and indicates that this treatment has the potential to disrupt SOD1 structure and expose buried epitopes (Additional file 3: Figure S1). In addition to this technique we also tested the immunoreactivity of C4F6 following steaming the tissue sections in citrate buffer for 30 minutes, which is a commonly used antigen retrieval technique. Tissue from hSOD1 non-transgenic mice displayed little or no immunoreactivity for C4F6 regardless of treatment (Figure 3a-c). In untreated tissue from mice expressing the G93A variant of hSOD1, to which the antibody was produced, strong C4F6 immunoreactivity was observed in the neuropil, including staining around the vacuolar structures that are commonly present in this line of mice at endstage (Figure 3d). The pattern and intensity of reactivity was changed little by treatment with formic acid and GdnHCl (Figure 3e). For tissues steamed in citrate buffer, the major difference observed was increased intra-neuronal staining in addition to the neuropil reactivity. In untreated tissue from mice overexpressing WT hSOD1 at ~18 months of age, rare punctate C4F6 immunoreactivity was observed in the neuropil and white matter (Figure 3g). Following either denaturation protocol, C4F6 immunoreactivity greatly increased in the neuropil in addition to staining around the rim of vacuolar deposits, identical to those found in G93A tissue from sick mice (Figure 3h,i). Treatment with citrate buffer seemed to slightly enhance the overall staining when compared to GdnHCl treatment while also enhancing neuronal cell body staining.

The evidence that the C4F6 antibody recognizes a conformational epitope derives from its high reactivity to fALS mutant SOD1 encoding other disease-associated mutations; a property that we observe in fixed, but otherwise untreated, transfected cultured cells [27]. In our hands, C4F6 staining of spinal cords from paralyzed G37R mice was relatively weak with only diffuse staining in the neuropil and occasional staining of cells resembling astrocytes (Figure 3j). In these mice, the overall level of C4F6 staining was greatly increased following either antigen retrieval technique, with increased staining in neuropil, robust neuronal cell body staining, and vacuolar staining observed in the gray matter (Figure 3k and l). Immunoreactivity of C4F6 to spinal cords of paralyzed mice expressing the truncated hSOD1 mutant L126Z was evident, but limited, in untreated tissues, but appeared to increase in intensity following either antigen retrieval technique (Figure 3m-o). In these L126Z mice, the antibody appeared to stain intra-cellular fibrillar structures as well as fibrillar structures in the neuropil. This model showed very little of the diffuse neuropil staining that was observed in tissue with the other SOD1 variants; a finding consistent with previous observations that this mutation is short-lived and primarily accumulates only in aggregates [21]. Taken together, these data reveal that antigen retrieval treatments that could potentially denature SOD1 produce C4F6 immunoreactivity for every SOD1 variant tested, including over-expressed WT hSOD1.

**Figure 4:**
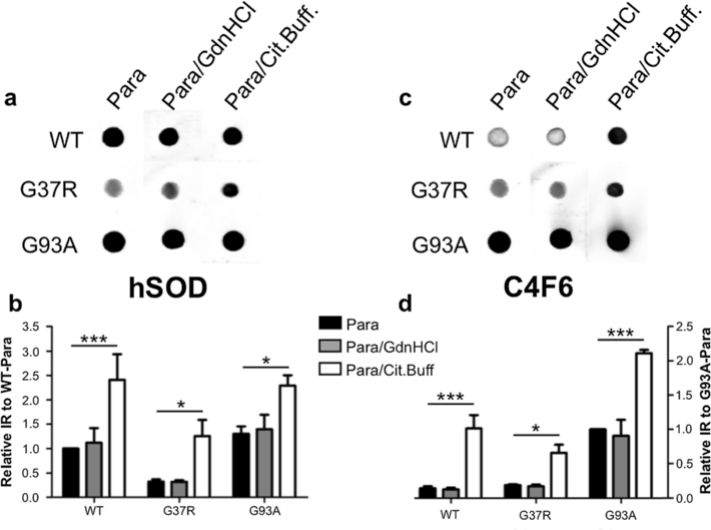
**Condition-specific immunoreactivity of the hSOD and C4F6 antibodies to purified recombinant hSOD1**. **(a)** 1.5 μg of the indicated recombinant hSOD1 protein was spotted onto a nitrocellulose membrane. All membranes were treated with 4% paraformaldehyde followed by either no treatment, 6 M guanidine hydrochloride (Para/GdnHCl), or steamed in citrate buffer (Para/Cit.Buff.) as described in Methods. Membranes were then blotted with the hSOD **(a)** and C4F6 **(c)** antibodies. **(b** and **d)** The intensities of the spots were quantified using an Odyssy Imaging System with the values normalized to the WT signal, for the hSOD blots, or to the G93A signal, for the C4F6 blots; the data from 3 independent experiments are graphed (mean ratio ± S.E. (error bars)). * P ≤ 0.05, *** P ≤ 0.001 (unpaired *t*-test).

To confirm the effects the two antigen retrieval techniques had on the affinity of C4F6 for the hSOD1 protein, we spotted purified, recombinant WT, G37R, and G93A hSOD1 proteins onto nitrocellulose, and fixed them with paraformaldehyde. The membranes were then treated with GdnHCl or citrate with steam, before testing immunoreactivity to C4F6. As a control, we also tested the immunoreactivity of these proteins with the hSOD antibody. In previous work, we have determined that this antibody cannot immunoprecipitate natively folded WT SOD1, but is highly reactive to WT SOD1 in fixed tissues [32] and to WT SOD1 over-expressed in cultured cells [27]. Following treatment of the nitrocellulose membrane with 6 M GdnHCl, we found the immunoreactivity for both hSOD and C4F6 antibodies was not significantly altered for any of the hSOD1 proteins tested (Figure 4a-d). This finding indicates that GdnHCl alone is not sufficient to denature the protein. Unfortunately, the combination of formic acid with GdnHCl could not be used because it dissolves nitrocellulose, and thus we cannot easily assay whether formic acid treatment with GdnHCl efficiently denatures SOD1. When the membrane was steamed in citrate buffer, the immunoreactivity of all three proteins was significantly increased for both hSOD and C4F6 antbodies. This finding indicates that the combination of heat and citrate buffer efficiently exposes the epitope for hSOD1 antibody (a peptide antibody) and similarly exposes an epitope recognized by C4F6.

### C4F6 immunoreactivity to misfolded WT SOD1 in postmortem tissue from sALS cases

**Figure 5:**
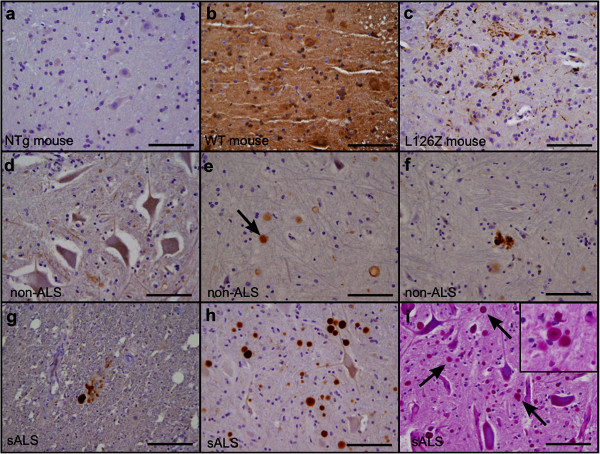
**Lack of C4F6 immunoreactivity in tissue from sALS cases.** For a point of reference, tissues from non-transgenic mice **(a)**, WT **(b)**, and L126Z **(c)** hSOD1 overexpressing mice were co-stained. These tissues were steamed in citrate buffer to enhance C4F6 immunoreactivity as described in Figure 5. **(d-f)** C4F6 staining of human tissue from non-disease controls revealed occasional spheroid (arrow in panel e) and punctate immunoreactive deposits (3 cases shown). **(g, h)** Similar structures were observed in human tissue from sALS cases (2 cases shown). For both the control and sALS cases the images shown are from tissues steamed in citrate. Less staining was observed in untreated tissues (Additional file 4: Figure S3). **(i)** The spheroid deposits were identical in appearance and frequency to structures identified as corpora amylacea (highlighted with arrows) by the Periodic Acid-Schiff stain. *Scale bar* 100 μm.

As previously discussed, two recent studies have investigated whether WT SOD1 in spinal motor neurons of sALS cases becomes reactive to the C4F6 antibody, producing data that was not entirely comparable because one study omitted antigen retrieval whereas the other used citrate/heat treatments [5, 15]. Having now achieved a better understanding of the nature of the epitope and the effects of antigen retrieval on antibody binding, we performed C4F6 immunohistochemistry on postmortem spinal cord tissue from a set of 25 sALS patients and 5 controls. We directly compared the two immunohistochemical protocols: either no antigen retrieval or steaming of the sections in citrate buffer that were used by Bosco et al. and Brotherton et al. [5, 15]. In our hands and in our patient samples, no significant difference was seen in the pattern of C4F6 staining whether the tissues were untreated prior to staining (Additional file 4: Figure S3) or steamed in citrate (Figure 5). As noted above, citrate treatment of mutant SOD1 mice clearly augmented reactivity to pathologic accumulations of mutant SOD1 in mice and hence we show images of the citrate treated human tissues for comparison. As controls for C4F6 staining, we used murine tissue from non-transgenic or hSOD1 over-expressing mice (Figure 5a-c). In both sALS and non-ALS human tissue examined, spherical C4F6-immunoreactive structures were observed (Figure 5d-f). These spheroids, which occasionally showed an immunopositive ring around a clear center, were found extracellularly throughout the gray and white matter of the spinal cord sections to varying degrees (Figure 5e-h). Occasionally, we observed extracellular punctate staining in the gray matter (Figure 5f,g). Eighteen of the 25 sALS cases and all five of the control cases we examined possessed these C4F6-reactive spheroids (Additional file 1: Table S1). Although the majority of cases revealed massive neurodegeneration and motor neuron loss, those tissue sections that retained motor neurons were devoid of intracellular C4F6 immunoreactivity. The regular shape of the spheroid structures was reminiscent of a common feature of adult human tissues termed corpora amylacea [33]. To determine the frequency of this pathology in our tissues, we performed a periodic acid Schiff stain, which is used for the detection of these deposits [33]. This stain revealed that these structures were frequent in our set of cases with the location and shape of the structure being identical to the C4F6 immunopositive spheroid deposits. Importantly, our control cases also had corpora amylacea deposits and the frequency of these deposits aligned with the frequency of C4F6 reactive spheroids in our control cases. Therefore, we concluded that the spheroid inclusion-like reactivity with C4F6 was either some type of cross reactivity with the corpora amylacea deposits or some non-disease related association of SOD1 with these structures. In any case, the structures were not specific to sALS cases.

## Discussion

In this study we sought to more precisely define the epitope of the anti-hSOD1 C4F6 antibody and elucidate the basis for its ability to specifically recognize many different ALS mutants of SOD1. Previous studies had determined that the epitope of C4F6 is located in exon 4 of the hSOD1 protein, which encompasses amino acids 80 to 119 [5]. Here, we used approaches in immunocytochemistry and immunoblotting to demonstrate that residues 90–93 of the protein (DKDG) comprise, at least in part, a critical element of the epitope recognized by C4F6. The importance of the Asp residue at position 90 was confirmed by demonstrating low immunoreactivity of C4F6 for the D90A variant of hSOD1 (either in fixed cells or on immunoblots). When C4F6 is presented with denatured protein on immunoblots, a much higher affinity of the antibody for SOD1 containing the D-K-D-A sequence at 90–93 (as would be the case for the G93A variant) over D-A-D-G (for WT hSOD1) was revealed. Thus, our data strongly implicate amino acids 90 and 93 of the hSOD1 protein as essential elements of the epitope recognized by C4F6 with residues D at amino acids 90 and G/A at 93 comprising the minimal residues responsible for antibody binding.

Structurally, amino acids 90–93 are located in the beta-turn structure formed by the loop between the 5^th^ and 6^th^ beta strands of hSOD1. An alignment of 32 distinct WT hSOD1 subunits from the PDB database of 7 independent crystal structures, revealed the consistent features of the protein’s conformation. The tertiary structure of WT hSOD1 brings the 90–93 loop in close proximity to the glutamic acid at position 40, which sits at the apex of the loop between beta strands 3 and 4. This region of the protein has been referred to as the β-plug because L38 caps one end of the β-barrel structure [34, 35]. At the apex of this plug, the side chain of Lys 91 shows two major populations; in one orientation of the side chain towards Asp 92 and in a less populated orientation toward Glu 40. The later case would favor hydrogen bonding between the amino group of Lys 91 and the carbonyl of the Glu residue at position 40. Additionally, due to their close proximity, numerous van der Waals interactions between these two loop structures would stabilize the native conformation regardless of whether the side chain of Lys 91 is positioned over Glu 40 or Asp 92. Collectively, these non-covalent interactions between the two loops stabilize a tight bend in the backbone of the protein to form the loop containing amino acids 90–93. We propose that in the structure of the WT native protein, the rigidity of this structure most likely prevents the C4F6 antibody from binding to an epitope that consists minimally of residues D-K-D-G/A at acids 90–93.

The structures available for SOD1-G93A (3 distinct structures with 16 subunits) show that residues 90–93 are generally found in a similar conformation as observed in WT SOD1. Thus, in comparing the WT and G93A variants in the region that contains a critical component of the epitope there is not an obvious conformational signature that we see as providing specificity for C4F6 binding. The C4F6 antibody was raised against recombinant apo G93A protein, the structure of which was included in the structures we analyzed and which completely aligns with that of metallated G93A protein. Thus, the conformational element caused by the G93A mutation that enables C4F6 binding does not appear to be due to a stable change in local structure around the 90–93 loop.

In studies of WT and mutant SOD1 expressed in cultured cells, our studies reported here, and previously [27], indicate that the C4F6 antibody can be used to detect misfolded mutant SOD1 and that most if not all fALS mutants share a common misfolded conformation around the loop between 90–93. By some manner mutations distant from the 90–93 loop, such as A4V [27], produce a structural change that causes amino acids 90–93 to adopt a conformation that enables the binding of C4F6 despite the lack of the favored A at position 93. NMR studies of the G93A mutant have provided evidence for local changes in protein structure, with the mutation causing amino acids D90 and V94 to have a much higher exposure to solvent than exists in the WT protein [36, 37]. Molecular-dynamic simulations of the A4V, G37R, and H46R variants of SOD1 have suggested destabilization of the β-plug region, which includes the 90–93 loop [38, 39]. Thus, multiple mutations can induce destabilization of the 90–93 loop and may explain why C4F6 shows greater reactivity to mutant SOD1 over WT protein when used for immunocytochemistry on transiently transfected cells [27]. One possible explanation for the ability of C4F6 to recognize many different mutants of SOD1 with greater avidity is that many mutations increase mobility in critical elements of the structure and this increased flexibility is propagated through the backbone to reduce the rigidity of the β-plug and allow the antibody greater access to the weaker epitope of D-K-D-G.

In regard to the conformational specificity of C4F6, our data indicate that the condition in which we have the greatest confidence that non-native conformation of the mutant SOD1 is driving reactivity is when used for immunocytochemistry in cell models. In our studies of cells over-expressing human SOD1 encoding ALS mutations other than G93A [27], the antibody consistently recognizes a subset of cells that over-express mutant SOD1. In these studies, cells are fixed briefly in paraformaldehyde and no particular antigen retrieval is required. Overall, our experience with the cell models suggests that C4F6 can discriminate between WT and mutant SOD1 by immunocytochemistry of fixed cells.

To determine the utility of the antibody in fixed tissues and the effect of common antigen retrieval procedures on binding, we examined C4F6 reactivity to spinal cords from mice overexpressing WT and mutant human SOD1. Treatments that we assumed would denature SOD1, treatment in formic acid and 6 M GdnHCl or heat treatment in citrate buffer, resulted in a significant increase in C4F6 staining in spinal cords from all SOD1 variants tested. Similarly, we observed increased C4F6 reactivity to purified SOD1 spotted onto nitrocellulose membrane after heat/citrate treatment. At least for the case in which heat/citrate treatment were used, we have confidence that some of the increased reactivity of the antibody to tissue is due to direct effects on the protein rather than facilitating better access of the antibody to its epitope. Because of the much greater affinity of the C4F6 antibody for the G93A protein, we cannot be sure whether the immunoreactivity detected in these mice is indicative of conformational change. Notably, in contrast to non-transgenic mice and mice expressing high levels of WT hSOD1, untreated spinal cords of paralyzed mice expressing the G37R and L126Z mutants showed at least some C4F6 immunoreactivity throughout the grey matter; either in the form of diffuse neuropil reactivity in the G37R mice or as discrete puncta in the L126Z mice. In all three mutant mouse models, antigen-retrieval treatments significantly increased the intensity of reactivity and, in the L126Z mice, elaborated fibrillar inclusions. However, these treatments also increased reactivity in the WT hSOD1 mice and thus we attribute C4F6 reactivity in this case to relaxation of the WT SOD1 structure to loosen the tight bend in the backbone around amino acids 90–93. Thus, although antigen retrieval methods do appear to enhance C4F6 binding to accumulations of SOD1 in pathologic structures, indicating the utility of the antibody, users should note that antigen retrieval methods that could denature SOD1 may produce C4F6 reactivity that is not necessarily indicative of the conformational state that existed in living tissue.

The C4F6 monoclonal antibody is one of several that have been used that were generated by immunizing mice with human G93A SOD1 [14, 40]. To determine whether our findings for C4F6 may extend to other monoclonal antibodies of this type, we examined the binding of the A5C3 monoclonal [40] to cells transfected with vectors to express WT and the G85R, G93A, and D90A variants (Additional file 5: Figure S2). Somewhat surprisingly, unlike C4F6, the A5C3 antibody showed no significant cross reactivity to G85R hSOD1 (Additional file 5: Figure S2). This finding suggests that the A5C3 antibody may have a greater dependency on the presence of an alanine at position 93 than the C4F6 antibody.

As described in the Introduction, there have been two prior studies of whether spinal motor neurons from cases of sALS show specific immunoreactivity to C4F6 antibody [5, 15]. One of these studies did not use antigen retrieval (Bosco et al.) while the other used a heat/citrate protocol similar to what we describe here (Brotherton et al.). In the Bosco et al. study, they demonstrated that a subset of motor neurons in a subset of cases (4 of 9) showed aberrant C4F6 immunoreactivity of spinal motor neuron cell bodies [5]. In the Brotherton et al. study, which compared sALS cases to an A4V fALS case and controls, C4F6 recognized skein like inclusions (similar to what we observed in the L126Z mouse model here) in the spinal motor neurons of A4V fALS cases. In sALS cases, as well as controls, no C4F6 reactive inclusions were observed and instead a more diffuse cell body staining was observed. In our hands and in our patient samples, whether tissues were stained without antigen retrieval or after heat/citrate treatment, the pattern of immunoreactivity in spinal cords of sALS and control patients were similar. In both, we observed that the most immunoreactive structures were extracellular spheroid and punctate deposits. No staining of motor neuron cell bodies was observed that resembled the immunoreactive cell bodies identified as containing misfolded hSOD1 in the Bosco study [5]. We noted a direct correlation between the frequency of C4F6 reactive spheroid structures and the frequency of corpora amylacea deposits, which are common glycoproteinacous inclusions that are frequently found in the CNS tissues of elderly people [33, 41]. These structures immunostain for multiple proteins including ubiquitin, heme oxygenase, Mn SOD1, and a-synuclein [42]. Whether the SOD1 reactivity present in these structures is indicative of a biologically relevant concentration of the protein or is some non-specific interaction with other components of the structure is unknown. The same C4F6 reactive structures were observed in non-ALS patients and thus these structures do not represent a disease-specific pathology. Thus, whether using antigen retrieval, which could reveal pathologic accumulations of SOD1, or no antigen retrieval, which could reveal SOD1 possessing a conformational signature produced by ALS mutant SOD1, we failed to detect robust C4F6 reactivity that is specific to sALS spinal motor neurons. Our data suggest that WT human SOD1 in spinal motor neurons in post-mortem tissues of sALS cases is infrequently present in a conformation that exposes the C4F6 epitope.

## Electronic supplementary material

Additional file 1: Table S1: Human Tissue Characteristics. (DOCX 20 KB)

Additional file 2: Table S2: Details for SOD1 structural analysis. (DOCX 15 KB)

Additional file 3: Figure S1: Antigen-retrieval impacts SEDI immunoreactivity to hSOD1 in transgenic mouse spinal cord tissues. Tissue sections from mice overexpressing WT, G93A, and G37R, or non-transgenic mice for controls, were stained with SEDI following either no antigen retrieval or formic acid and 6M guanidine hydrochloride (FA & GdnHCl). All tissues from mice expressing mutant hSOD1 (G93A and G37R) were harvested from paralyzed mice. The WT hSOD1 tissue was from mice ~18 months of age. *Scale bar* 100 μm. (PDF 207 KB)

Additional file 4: Figure S3: C4F6 immunoreactivity in tissue from sALS cases without antigen retrieval. Tissues from nontransgenic mice **(a)**, WT **(b)**, and G93A **(c)** hSOD1 overexpressing mice were stained with C4F6 as controls. **(d)** C4F6 staining of human tissue from non-disease controls revealed very little immunoreactivity. **(e, f)** In human tissue from sALS cases (2 cases shown), occasional spheroid deposits appeared to be C4F6 immunopositive. For both the control and sALS cases the images shown are from tissues not treated for antigen retrieval. The spheroid deposits were identical in appearance and frequency to structures identified as corpora amylacea (highlighted with arrows) by the Periodic Acid-Schiff stain. *Scale bar* 200 μm. (PDF 40 MB)

Additional file 5: Figure S2: C4F6 and A5C3 differ in their specificity for SOD1. C4F6 revealed no immunoreactivity to WT SOD1 **(c)** but stained cells transfected with G85R SOD1 **(d)**. Following transient transfection with WT **(e, i)**, G93A **(f, j)**, G85R **(g, k)**, or D90A **(h, l)** cells immunostained with the A5C3 antibody only showed reactivity to G93A **(j)**. Nuclei were stained with DAPI (blue). *Scale bar* 50 μm. (PDF 5 MB)
