# Supplementary material for: Conformational specificity of the C4F6 SOD1 antibody; low frequency of reactivity in sporadic ALS cases
Source: Acta Neuropathol Commun. 2014 May 14;2:55. doi: 10.1186/2051-5960-2-55 (PMC4035506; doi:10.1186/2051-5960-2-55)
Supplement: Supplementary file 1 — Additional file 1: Table S1: Human Tissue Characteristics. (DOCX 20 KB) [file 40478_2014_128_MOESM1_ESM.docx]

| **Case** | **Age at death** | **Sex** | **Motor neuron loss^a^** | **Bunina bodies^a^** | **Cystain C Neuron^a^** | **Skein-like inclusions^a^** | **Lewy-body-like inclusions^a^** | **PAS+ Corpora amylacea^b^** |
| --- | --- | --- | --- | --- | --- | --- | --- | --- |
| ALS-1 | 68 | F | + | + | + | +++ | +++ | - |
| ALS-2 | 56 | M | +++ | - | +/- | - | - | - |
| ALS-3 | 51 | M | + | + | + | +++ | + | + |
| ALS-4 | 57 | M | ++ | - | +/- | + | - | - |
| ALS-5 | 59 | F | + | n/a^c^ | + | + | + | + |
| ALS-6 | 80 | M | + | - | +/- | - | - | + |
| ALS-7 | 50 | F | ++ | - | - | + | + | - |
| ALS-8 | 70 | F | +++ | -? | + | + | - | - |
| ALS-9 | 55 | F | ++ | +? | - | ++ | + | + |
| ALS-10 | 60 | M | ++ | - | - | - | - | - |
| ALS-11 | 57 | F | + | ++ | - | ++ | ++ | + |
| ALS-12 | 64 | F | + | ++ | ++ | +++ | + | + |
| ALS-13 | 79 | M | +++ | - | - | - | - | + |
| ALS-14 | 67 | F | +++ | - | - | + | + | - |
| ALS-15 | 68 | M | + | + | ++ | + | + | - |
| ALS-16 | 61 | F | + | - | - | - | - | + |
| ALS-17 | 53 | M | ++ | - | +/- | - | - | - |
| ALS-18 | 79 | M | ++ | n/a | + | ++ | ++ | + |
| ALS-19 | 63 | F | ++ | + | + | + | - | - |
| ALS-20 | 61 | F | + | ++ | + | - | + | + |
| ALS-21 | 77 | F | + | +++ | +++ | + | +++ | - |
| ALS-22 | 71 | M | + | - | - | - | - | - |
| ALS-23 | 42 | M | ++ | - | - | - | - | + |
| ALS-24 | 54 | F | ++++ | - | - | + | - | + |
| ALS-25 | 55 | M | + | - | - | ++ | - | + |
| Cntrl-1 | 43 | M | - | n/a | n/a | n/a | n/a | + |
| Cntrl-2 | 46 | F | - | n/a | n/a | n/a | n/a | + |
| Cntrl-3 | 42 | M | - | n/a | n/a | n/a | n/a | + |
| Cntrl-4 | 50 | M | - | n/a | n/a | n/a | n/a | + |
| Cntrl-5 | 48 | M | - | n/a | n/a | n/a | n/a | + |

**Additional file 1: Table S1. Human Tissue Characteristics**

^a^ semi-quantitative assessment of abundance

^b^ presence (+) or absence (-)

^c^ not available
