# Supplementary material for: Conformational specificity of the C4F6 SOD1 antibody; low frequency of reactivity in sporadic ALS cases
Source: Acta Neuropathol Commun. 2014 May 14;2:55. doi: 10.1186/2051-5960-2-55 (PMC4035506; doi:10.1186/2051-5960-2-55)
Supplement: Supplementary file 2 — Additional file 2: Table S2: Details for SOD1 structural analysis. (DOCX 15 KB) [file 40478_2014_128_MOESM2_ESM.docx]

**Additional file 2: Table S2. Details for SOD1 structural analysis**

|  | Description | PDB Code | Reference | Resolution | #monomers/  AU |
| --- | --- | --- | --- | --- | --- |
| 1* | WT | 1hl5 | JMB2003 PMID  12729761 | 1.8 | 18 |
| 2* | WT | 3ecu | PNAS  PMID 19369197 | 1.9 | 4 |
| 3 | WT | 1hl4 | JMB2003  PMID 12729761 | 1.8 | 2 |
| 4 | WT | 2c9u | JMB2006  PMID 16406071 | 1.24 | 2 |
| 5 | WT | 2c9v | JMB2006  PMID 16406071 | 1.1 | 2 |
| 6 | WT | 2v0a | PNAS2007  PMID 17548825 | 1.15 | 2 |
| 7 | WT | 2c9s | JMB2006  PMID 16406071 | 1.24 | 2 |
| 8 | G93A | 3gzo | ABB2009  PMID  19800308 | 2.1 | 10 |
| 9 | G93A | 3gzp | ABB2009  PMID  19800308 | 3.1 | 4 |
| 10 | G93A | 2wko | ABB2009  PMID  19800308 | 1.97 | 2 |
