# Supplementary material for: Conformational specificity of the C4F6 SOD1 antibody; low frequency of reactivity in sporadic ALS cases
Source: Acta Neuropathol Commun. 2014 May 14;2:55. doi: 10.1186/2051-5960-2-55 (PMC4035506; doi:10.1186/2051-5960-2-55)
Supplement: Supplementary file 3 — Additional file 3: Figure S1: Antigen-retrieval impacts SEDI immunoreactivity to hSOD1 in transgenic mouse spinal cord tissues. Tissue sections from mice overexpressing WT, G93A, and G37R, or non-transgenic mice for controls, were stained with SEDI following either no antigen retrieval or formic acid and 6M guanidine hydrochloride (FA & GdnHCl). All tissues from mice expressing mutant hSOD1 (G93A and G37R) were harvested from paralyzed mice. The WT hSOD1 tissue was from mice ~18 months of age. Scale bar 100 μm. (PDF 207 KB) [file 40478_2014_128_MOESM3_ESM.pdf]

Electronic Supplementary Material 3

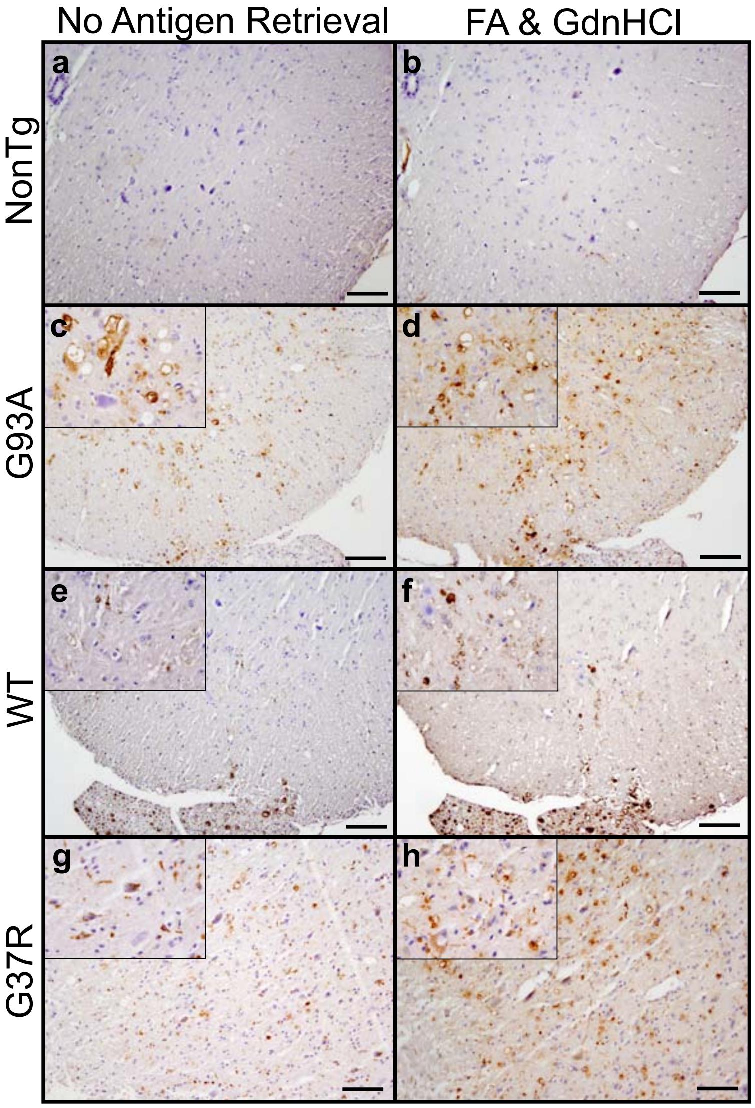

ESM3 Antigen-retrieval impacts SEDI immunoreactivity to hSOD1 in transgenic mouse spinal cord tissues. Tissue sections from mice overexpressing WT, G93A, and G37R, or non-transgenic mice for controls, were stained with SEDI following either no antigen retrieval or formic acid and 6M guanidine hydrochloride (FA & GdnHCl). All tissues from mice expressing mutant hSOD1 (G93A and G37R) were harvested from paralyzed mice. The WT hSOD1 tissue was from mice ~18 months of age. *Scale bar* 100 µm
