# Supplementary material for: Conformational specificity of the C4F6 SOD1 antibody; low frequency of reactivity in sporadic ALS cases
Source: Acta Neuropathol Commun. 2014 May 14;2:55. doi: 10.1186/2051-5960-2-55 (PMC4035506; doi:10.1186/2051-5960-2-55)
Supplement: Supplementary file 4 — Additional file 4: Figure S3: C4F6 immunoreactivity in tissue from sALS cases without antigen retrieval. Tissues from nontransgenic mice (a), WT (b), and G93A (c) hSOD1 overexpressing mice were stained with C4F6 as controls. (d) C4F6 staining of human tissue from non-disease controls revealed very little immunoreactivity. (e, f) In human tissue from sALS cases (2 cases shown), occasional spheroid deposits appeared to be C4F6 immunopositive. For both the control and sALS cases the images shown are from tissues not treated for antigen retrieval. The spheroid deposits were identical in appearance and frequency to structures identified as corpora amylacea (highlighted with arrows) by the Periodic Acid-Schiff stain. Scale bar 200 μm. (PDF 40 MB) [file 40478_2014_128_MOESM4_ESM.pdf]

Supplementary Figure 3

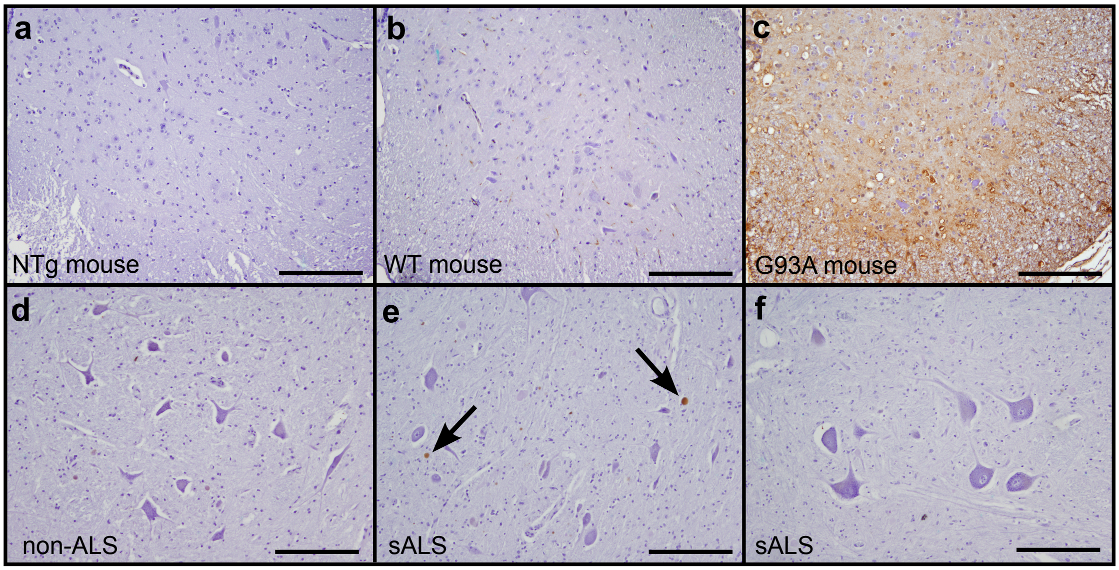

Figure S3 C4F6 immunoreactivity in tissue from sALS cases without antigen retrieval. Tissues from non-transgenic mice (**a**), WT (**b**), and G93A (**c**) hSOD1 overexpressing mice were stained with C4F6 as controls. (**d**) C4F6 staining of human tissue from non-disease controls revealed very little immunoreactivity. (**e, f**) In human tissue from sALS cases (2 cases shown), occasional spheroid deposits appeared to be C4F6 immunopositive. For both the control and sALS cases the images shown are from tissues not treated for antigen retrieval. The spheroid deposits were identical in appearance and frequency to structures identified as corpora amylacea (highlighted with arrows) by the Periodic Acid-Schiff stain. *Scale bar* 200 µm
