# Supplementary material for: Conformational specificity of the C4F6 SOD1 antibody; low frequency of reactivity in sporadic ALS cases
Source: Acta Neuropathol Commun. 2014 May 14;2:55. doi: 10.1186/2051-5960-2-55 (PMC4035506; doi:10.1186/2051-5960-2-55)
Supplement: Supplementary file 5 — Additional file 5: Figure S2: C4F6 and A5C3 differ in their specificity for SOD1. C4F6 revealed no immunoreactivity to WT SOD1 (c) but stained cells transfected with G85R SOD1 (d). Following transient transfection with WT (e, i), G93A (f, j), G85R (g, k), or D90A (h, l) cells immunostained with the A5C3 antibody only showed reactivity to G93A (j). Nuclei were stained with DAPI (blue). Scale bar 50 μm. (PDF 5 MB) [file 40478_2014_128_MOESM5_ESM.pdf]

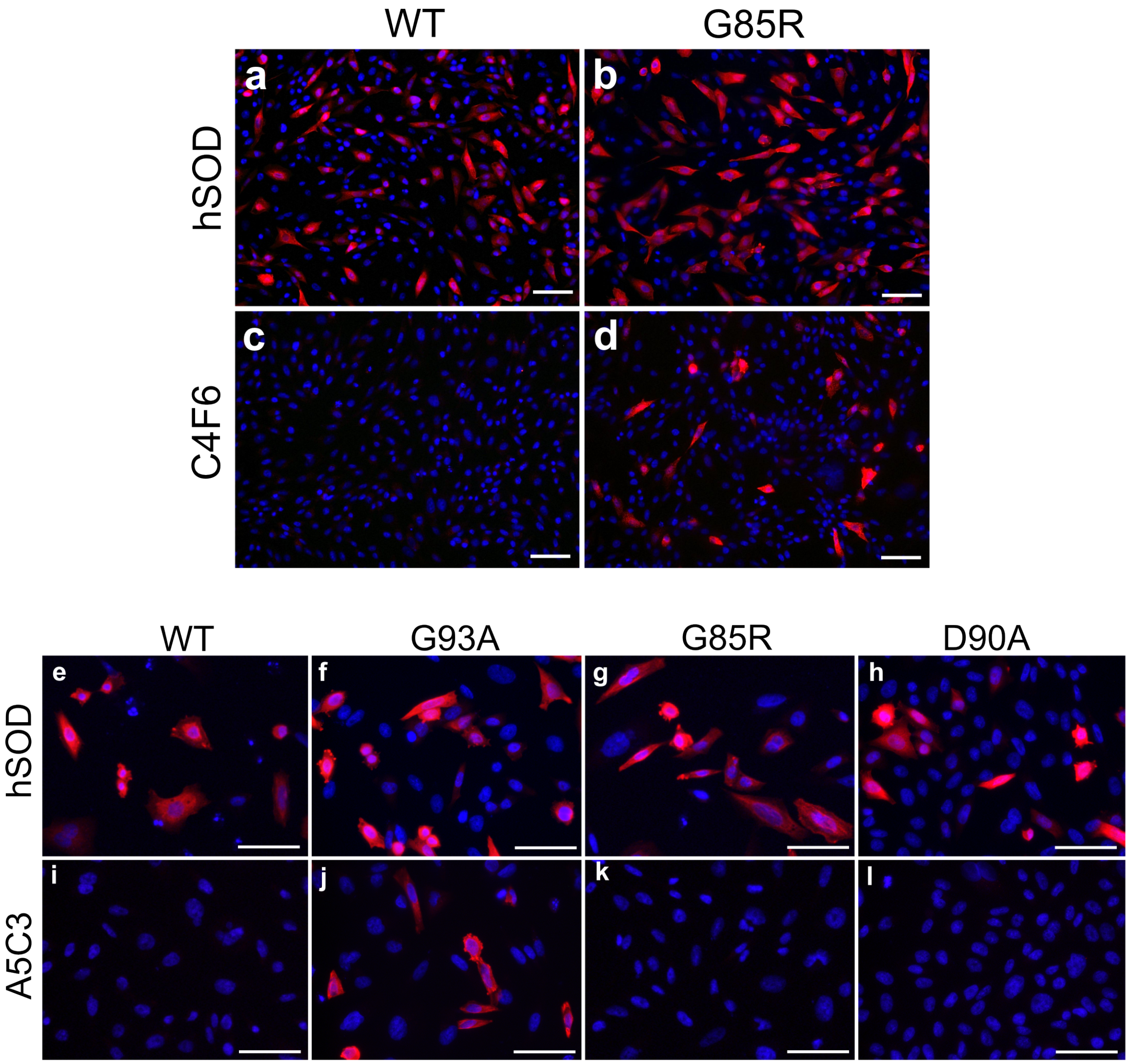


**ESM2** C4F6 and A5C3 differ in their specificity for SOD1**.** C4F6 revealed no immunoreactivity to WT SOD1 (**c**) but stained cells transfected with G85R SOD1 (**d**). Following transient transfection with WT (**e, i**), G93A (**f, j**), G85R (**g, k**), or D90A (**h, l**) cells immunostained with the A5C3 antibody only showed reactivity to G93A (**j**). Nuclei were stained with DAPI (blue). *Scale bar* 50 µm.
